# Solar-Driven Hydrogen Peroxide Production Using Polymer-Supported Carbon Dots as Heterogeneous Catalyst

**DOI:** 10.1007/s40820-017-0143-7

**Published:** 2017-03-17

**Authors:** Satyabrat Gogoi, Niranjan Karak

**Affiliations:** Advanced Polymer and Nanomaterial Laboratory, Department of Chemical Sciences, Tezpur University, Tezpur, 784028 Assam India

**Keywords:** Carbon dot, Photo-catalyst, Heterogeneous catalyst, Hydrogen peroxide

## Abstract

Safe, sustainable, and green production of hydrogen peroxide is an exciting proposition due to the role of hydrogen peroxide as a green oxidant and energy carrier for fuel cells. The current work reports the development of carbon dot-impregnated waterborne hyperbranched polyurethane as a heterogeneous photo-catalyst for solar-driven production of hydrogen peroxide. The results reveal that the carbon dots possess a suitable band-gap of 2.98 eV, which facilitates effective splitting of both water and ethanol under solar irradiation. Inclusion of the carbon dots within the eco-friendly polymeric material ensures their catalytic activity and also provides a facile route for easy catalyst separation, especially from a solubilizing medium. The overall process was performed in accordance with the principles of green chemistry using bio-based precursors and aqueous medium. This work highlights the potential of carbon dots as an effective photo-catalyst.

**Electronic supplementary material:**

The online version of this article (doi:10.1007/s40820-017-0143-7) contains supplementary material, which is available to authorized users.

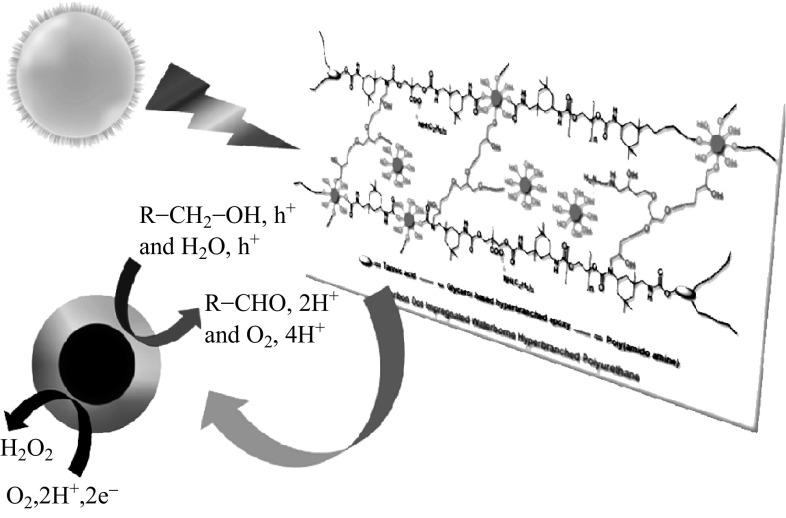

## Highlights

- Polyurethane-supported carbon dots were developed as heterogeneous catalyst, and the developed catalyst was biodegradable.
- Solar-driven H_2_O_2_ production from water, ethanol, and oxygen was achieved.

## Introduction

Recently, greener, inexpensive, and efficient water splitting techniques have garnered significant interest in the thrust toward sustainable energy conversion processes [1–3]. In this context, photo-catalysis has been adopted as a “green” and “sustainable” technique [4]. In the last few years, much effort has been devoted toward sustainable production of hydrogen peroxide (H_2_O_2_) by photo-splitting of water (H_2_O). In the current scenario of rapid depletion of fossil fuels, sustainable production of H_2_O_2_ is very significant. H_2_O_2_ can be used as a fuel in one-compartment fuel cells with an output potential of 1.09 V, which is comparable to that of the hydrogen fuel cell with an output value of 1.23 V [5]. Further, H_2_O_2_ serves as a clean and green oxidant with H_2_O as the only by-product [6]. Thus, green and sustainable production of H_2_O_2_ is highly desirable for implementation of “green chemistry” principles and can provide a promising prospect for resolving global fuel woes.

In the conventional process, H_2_O_2_ is produced by the anthraquinone method, which is highly disadvantageous from the green chemistry point of view. The drawbacks include the multi-step process, and high energy consumption, et cetera [7]. Catalytic systems such as Pd and bi-metallic Au–Pd have been studied for the direct production of H_2_O_2_ from H_2_ and O_2_ [8–11]. Such direct methods are effective for quantitative production of H_2_O_2_, but have an attendant high risk because of the explosive nature of the H_2_/O_2_ mixture. Most recently, semiconductor-based nanocatalysts have been used for sustainable production of H_2_O_2_ from H_2_O and alcohols. Such methods involve irradiation of an oxygen (O_2_)-saturated alcohol or aqueous system under UV light in the presence of metallic semiconductors. State-of-the-art literature cites the use of different metal-based semi-conductor catalysts for sustainable production of H_2_O_2_ from H_2_O and alcohol. These include Ru-complexes, Ir-complexes, Ti nanoparticles, and Ni, et cetera [12–20]. However, the major concerns associated with these catalysts are their high cost, toxicity, metal-based origin, and non-degradable nature. In an effort to introduce metal-free and degradable catalysts, Shiraishi and his group reported polymeric graphitic carbon nitride (*g*-C_3_N_4_) for the production of H_2_O_2_ from ethanol [21]. However, the high production cost and low band-gap of *g*-C_3_N_4_ hamper its successful use, especially for splitting H_2_O under solar irradiation. Thus, the development of an eco-friendly and sustainable catalyst system with the capacity to produce H_2_O_2_ is desirable to meet the current challenges.

Carbon dots (CDs), with unique physical, chemical, and optical properties, have attracted immense interest in the scientific community based on their utility in diverse areas, such as bio-imaging, biosensors, light-emitting diodes, and other opto-electrical devices [22–25]. The unique traits of CDs include their excitation wavelength-dependent stable multi-color emission and excellent aqueous solubility with remarkable cytocompatibility. Like other quantum dots, exhibition of the “quantum confinement” effect is the typical signature of CDs. CDs consist predominantly of amorphous carbon along with *sp* ^*2*^ hybridized graphitic carbon. The most fascinating feature of CDs is their capability to harvest photons in the UV/visible spectral region. Such light-harvesting ability renders CDs as a potential candidate for photo-catalytic application [1, 26]. However, in practice, there are certain difficulties in the use of bare CDs as an effective photo-catalyst. CDs are soluble in water or organic solvents like ethanol. This creates serious difficulties in their separation when such media are used. However, in media where CDs are insoluble, CDs suffer solid-state quenching of the photo-activity [27]. Such obstacles restrict the practical applicability of CDs as photo-catalysts.

In this context, the use of a polymeric matrix as a support can facilitate the application of CDs. The polymer matrix can prevent solid-state quenching and also provide sufficient mechanical strength and catalytic stability and facilitate application with easy separation. Such polymer/CD nanocomposite systems have found use in opto-electrical applications. Photo-catalytic applications have also been reported recently [28]. In this regard, we found that the waterborne hyperbranched polyurethane (WPU) system is attractive as a polymer support. The solubility of both the nanomaterial and polymer in the same medium (aqueous) expedites homogeneous distribution of the CDs over the polymer matrix, thereby preserving their photo-harvesting ability. Further, the use of WPU offers additional advantages. During the polymerization reaction, WPU becomes soluble due to the creation of ionic centers along the polymer chains. Thus, it behaves like an ionomer with electron transfer capability. Hence, compared to conventional polymeric systems, WPU is expected to act as a better catalyst support for photo-catalytic reactions.

Herein, a CD-impregnated WPU nanocomposite film is used as a heterogeneous photo-catalyst for solar-driven production of H_2_O_2_ by using H_2_O, C_2_H_5_OH, and O_2_ as raw materials. Further, bacterial degradation of the catalyst is carried out by using *Pseudomonas aeruginosa* to highlight its eco-friendly behavior. Thus, the present study reports a metal-free, low-cost, easily separable, non-toxic, and biodegradable carbon dot-impregnated polymeric nanocomposite system as a heterogeneous catalyst for sustainable and green production of H_2_O_2_.

## Experimental

### Materials

Isophorone diisocyanate (IPDI, Aldrich, Germany), poly(ethylene glycol) with a number average molecular weight (*M*_*n*_) of 600 g mol^−1^ (PEG 600, Merck, India), 2,2-bis(hydroxymethyl) propionic acid (BMPA, Aldrich, Germany), 1,4-butanediol (BD, Merck, India), tannic acid (TA, Sigma-Aldrich, Belgium), triethylamine (TEA, Merck, India), and tetrahydrofuran (THF, Merck, India) were used for WPU preparation. Glycerol-based hyperbranched epoxy (HE) and vegetable oil-based poly(amide amine) (PAA) were used to cross-link the fabricated WPU/CD system [29, 30]. Corms of *Colocasia esculenta* were used to prepare the CDs. Moreover, ethanol (C_2_H_5_OH, Merck, India), Millipore water (H_2_O, obtained from MilliQ^®^ Ultrapure Water Solutions, Type 1, water purifier system), and oxygen (O_2_, obtained from Jainex Gas Company, India) were used as starting materials in the photo-catalytic application. Potassium permanganate (KMnO_4_, Merck, India) was used in the estimation of hydrogen peroxide by following a titrimetric method. In the biodegradation study, the bacterial strain *Pseudomonas aeruginosa* (strain MTCC 7814) was used. This strain was procured from the Microbial Type Culture Collection and Gene Bank, Chandigarh, India. The bacterial culture was collected through the Department of Molecular Biology and Biotechnology, Tezpur University, India.

### Catalyst Preparation

The CDs were prepared by using corms of *C. esculenta* as reported earlier [31]. However, a slight modification was made to the synthetic procedure. In order to exert greater control over the particle size and size distribution, a hydrothermal method was employed. Briefly, an aqueous extract (100 mL) of corms of *C. esculenta* was heated at 150 °C for 5 h in a Teflon-lined hydrothermal reactor (250 mL capacity). The CDs formed were dried under reduced pressure. The CD-impregnated WPU polymer was prepared via an ex situ technique according to a method described elsewhere [31]. However, in the present study, we used a high CD loading compared to that previously reported. Briefly, IPDI, PEG 600, and BD were reacted in the pre-polymerization step at a temperature of 85 ± 2 °C. In the next step, TA and BD were allowed to react with the pre-polymer formed in the first step at 70 ± 2 °C for 4–4.5 h. This process was followed by neutralization using TEA at room temperature. Finally, water was added and THF was removed under reduced pressure. Different weight percentages of CD were incorporated into the WPU matrix by using mechanical and ultra-sound energy. These nanocomposites were coded as PNC1.0, PNC2.0, PNC3.0, PNC4.0, and PNC5.0 for CD loadings of 1, 2, 3, 4, and 5 wt%, respectively. Further, the nanocomposites were cross-linked by using HE and PAA. Details of the fabrication technique are provided in Supplementary Information.

### Catalyst Characterization

X-ray diffraction (XRD) patterns of the CDs and CD-impregnated WPU were obtained by using a Bruker AXS, Germany, Model: D8 Focus instrument (2*θ* = 10°–70° at a scanning rate of 2°min^−1^). The graphitic structure of the CDs was studied using Raman spectroscopy (Renishaw, UK, Renishaw Basis Series with 514 nm Laser). The shape and size of the CDs, as well as the distribution of the CDs over the WPU matrix, were visually studied by using high-resolution transmission electron microscopic (HRTEM) analysis. TEM images were obtained by using a JEOL, JEMCXII, Japan, microscope at an operating voltage of 200 kV using a Cu grid (TED PELLA INC, Ultrathin C, Type A, 400 mesh). The microscopic data were analyzed as inverse fast Fourier transform (IFFT) images by using Gatan Digital Micrograph software. A UV–visible photo-spectrometer (Thermo-Scientific, Evolve 300, USA) was used to determine the band-gap. The photoluminescence (PL) behavior was studied by using a PerkinElmer, USA, Model: LS-55 fluorescence spectrometer. The surface morphology of the biodegraded catalyst was studied by scanning electron microscopy (SEM, JEOL, JSM 6390LV) after platinum coating the surface.

### Band-Gap

The band-gap of the CDs was evaluated by using UV–visible spectroscopy and applying Eq. 1 [32],

1 $$\alpha = C(h\nu - E_{\text{bulk}} )^{1/2} /h\nu$$

Here *α* is the absorption coefficient, *C* is a constant, h is Plank’s constant, *ν* is the frequency, and *E*_bulk_ is the energy gap. In practice, the band-gap is calculated by plotting h*ν* versus (*α*h*ν*)^2^. Extrapolation of the curve to (*α*h*ν*)^2^ gives the value of *E*_bulk_.

### Determination of Quantum Yield

The quantum yield of the CDs and PNCs was determined by using Eq. 2 [33].

2 $$Q_{\text{CD}} = Q_{\text{R}} \frac{{I_{\text{CD}} \cdot A_{\text{R}} \cdot \eta_{\text{CD}}^{2} }}{{I_{\text{R}} \cdot A_{\text{CD}} \cdot \eta_{\text{R}}^{2} }}$$

Here, *Q*_CD_ is the quantum yield of the CDs, *Q*_R_ is the quantum yield of the reference compound (quinine sulfate), *I*_CD_ is the intensity of the emission of the CDs, *I*_R_ is the intensity of the luminescence of the reference compound (quinine sulfate), *A*_CD_ is the absorbance of the CDs at the given excitation wavelength (360 nm), *A*_R_ is the absorbance of the reference compound (quinine sulfate) at the given excitation wavelength (360 nm), and *η* is the refractive index of the solvent used (water). The quantum yield of quinine sulfate at 360 nm is 54%.

### Photo-catalytic Reaction

Five different compositions, i.e., H_2_O, C_2_H_5_OH (100%), and mixtures of H_2_O and C_2_H_5_OH (25, 50, and 75% (v/v)) were used as the starting materials for the solar-driven production of H_2_O_2_. The reactants were placed into a two-necked glass reactor equipped with an O_2_ inlet. Initially, the mixture was saturated with molecular O_2_ by passing the gas for 10 min and the reactor was then sealed. The system was then irradiated under normal solar light with an intensity of 80,000–100,000 lx at ca. 25 °C with a continuous supply of O_2_ for different time intervals (5–50 h). The production of H_2_O_2_ was estimated via the redox titrimetric method using KMnO_4_ [23].

### Biodegradation Study

Analysis of the biodegradability of the catalyst system was carried out by using *P. aeruginosa* as the test organism [34]. A mineral salt solution containing 2.0 g (NH_4_)_2_SO_4_, 2.0 g Na_2_HPO_4_, 4.75 g KH_2_PO_4_, 1.2 g MgSO_4_·7H_2_O, 0.5 mg CaCl_2_·2H_2_O, 100 mg MnSO_4_·5H_2_O, 70 mg ZnSO_4_·7H_2_O, 10 mg H_3_BO_3_·5H_2_O, 100 mg CuSO_4_·7H_2_O, 1 mg FeSO_4_·7H_2_O, and 10 mg MoO_3_ in 1000 mL distilled water was prepared and sterilized at 120 °C under 15 lb pressure. The *P. aeruginosa* bacterial strain was cultured in a shaker incubator for 48 h using this medium. A 100-µL aliquot of the cultured bacteria was placed in a conical flask with 10 mL of the salt medium. UV-sterilized (wavelength 254 nm) catalyst films with a known weight were immersed in the medium and incubated at 37 °C for seven weeks. Growth of the bacteria was followed by measuring the absorbance of the medium at 600 nm at an interval of seven days. Similarly, a change in the weight of the catalyst was recorded after every seven days. SEM images of the biodegraded films were also taken after completion of the experimental period.

## Results and Discussion

The synthesis of the CDs provides a green and sustainable route in which a large number of naturally available carbon resources are utilized [35]. In the present study, a carbohydrate-rich aqueous extract of corms of *C. esculenta* was used as the precursor. The CDs were obtained through a hydrothermal method. It can be assumed that in the hydrothermal process, extensive carbonization and aromatization took place through condensation and polymerization of different carbohydrate molecules at elevated temperature and pressure [33, 36].

**Fig. 1:**
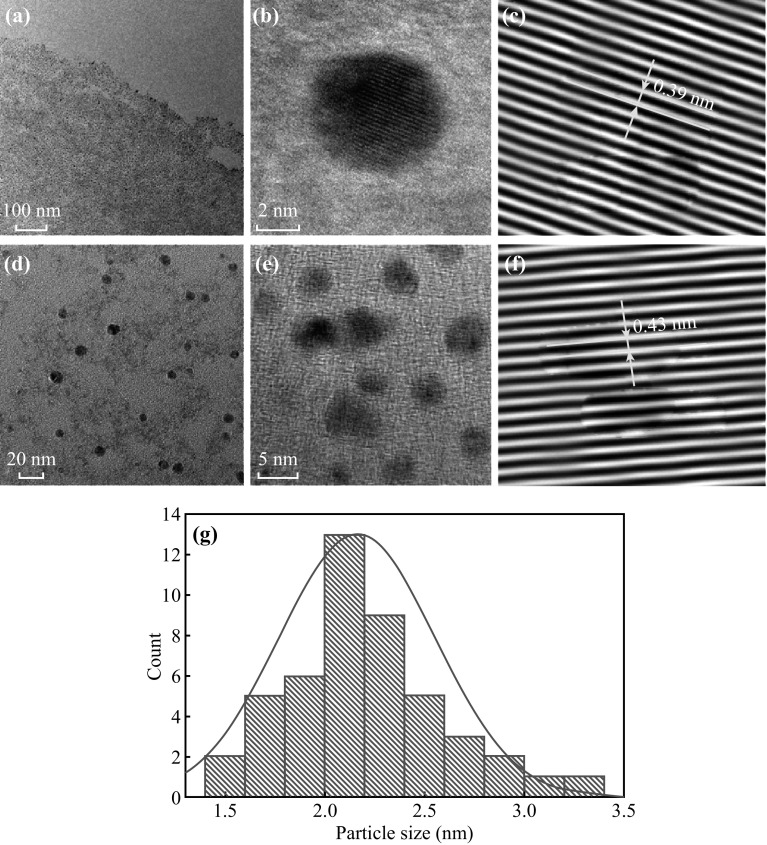
**a** TEM image of CD. **b** HRTEM of CD. **c** IFFT image of CD. **d** TEM image of PNC4.0. **e** HRTEM of PNC4.0. **f** IFFT of PNC4.0. **g** Size distribution of CDs

The prepared CDs were characterized by TEM, XRD, and Raman spectroscopic techniques. As shown in Fig. 1a, b, the TEM image of the CDs indicates the formation of carbon nanoparticles with nearly spherical morphology. Statistical evaluation of the size distribution revealed that the size of the CDs ranged from 1.5 to 3.5 nm. The majority of the CDs has sizes in the range of 1.7–2.1 nm (see Fig. 1g). Compared to the simple heating process (at 150 °C for 3.5 h) reported previously by the same group, the size distribution was found to be restricted within a narrow range and with a smaller diameter [31]. Controlling the size of the CDs is important because the size can influence the band-gap, which is a critical parameter for water splitting.

**Fig. 2:**
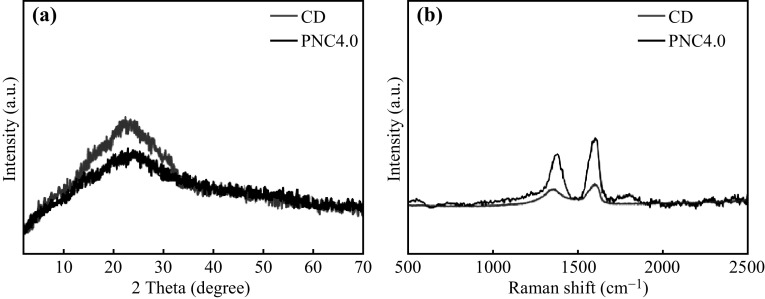
**a** XRD patterns and **b** Raman spectra of CDs and PNC4.0

Figure 1c shows the IFFT image of the CDs, indicating a layer spacing of 0.39 nm, which can be assigned to the (022) graphitic plane of the CDs. However, compared to the inter-planar distance of the pure graphitic structure (~0.34 nm), the spacing was greater in the case of the CDs. This may be ascribed to the presence of oxygeneous functionalities in the CD structure, which are intercalated between the consecutive layers, leading to a larger inter-planar seperation. In comparison, the XRD pattern of the CDs displayed a broad peak centered around 2*θ* = 22° (Fig. 2a). This is characteristic of a weakly crystalline graphitic structure with a large number of defect sites [33].

The Raman spectrum of the CDs displayed two distinct bands, i.e., the D band near 1360 cm^−1^ and the G band near 1580 cm^−1^ (Fig. 2b). The D band is characteristic of structural defects, and the G band is characteristic of symmetric *sp* ^2^ graphitic carbon stretching. UV–visible spectroscopy shows a peak near 270 nm, which is an indication of successful formation of the CDs (Fig. S1). Thus, TEM, XRD, Raman, and UV–visible spectroscopic analyses confirmed formation of the CDs. The CDs were incorporated into the WPU matrix for the development of a heterogeneous catalyst system for solar-driven H_2_O_2_ production.

**Fig. 3:**
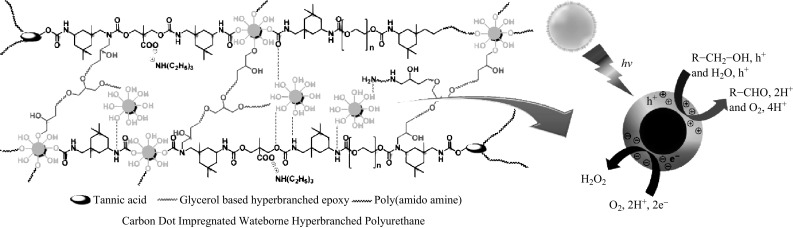
Solar-driven H_2_O_2_ production using CD-impregnated waterborne hyperbranched polyurethane as heterogeneous catalyst

In this context, the critical issue is to obtain a homogeneous distribution of the CDs over the WPU matrix without agglomeration. Agglomeration of the CDs can lead to the loss of their light-absorbing capability, which in turn hampers their photo-catalytic activity. However, in the present study, we found that the CDs and WPU acted complementarily to produce a compatible and uniform composite system. The CDs, being a highly functional nanomaterial, undergo sufficient interactions (both covalent and non-covalent) with the hyperbranched polymer matrix (Fig. 3). Such prominent interactions, along with the dispersibility of both the polymer and the nanomaterial in the reaction medium, helped to produce a homogeneous polymer-supported nanosystem without any agglomeration.

Visual evidence obtained from the TEM image of the catalyst system confirmed the uniformity, even at high nanomaterial loading (4 wt%) (Fig. 1d). Comparison of the HRTEM images (Fig. 1b, e) and corresponding IFFT images of the CDs and PNC4.0 (Fig. 1c, f) revealed an increase in the interlayer spacing from 0.39 nm for the pristine CDs to 0.43 nm after incorporation into the polymer matrix. This may be due to the additional defects created within the CD structure during the fabrication process. Intercalation of the polymeric chains may also increase the interlayer distance. This indicates strong interaction between the polymer and nanomaterial.

The XRD pattern of PNC4.0 showed a peak corresponding to amorphous carbon near 2*θ* = 22°, ascribed to the (002) plane of graphitic carbon (Fig. 2a). Similar to the profile of the CDs, the Raman spectrum of PNC4.0 displayed D and G bands, albeit with a higher *I*_D_/*I*_G_ ratio. This provides evidence of the generation of defects in the CDs during the fabrication process. On the other hand, modification of this system with HE and PAA is expected to provide better catalyst stability and prevent CD leaching by the formation of a cross-linked network structure.

Before studying the photo-catalytic activity of the prepared catalyst, effort was made to evaluate different parameters, which ensured suitability of the polymer-supported CD system as a photo-catalyst for H_2_O splitting. By virtue of their quantum dimension, the CDs possess the capability to harvest photons over the solar spectrum (covering the entire UV–visible spectral region, extending to the near IR) based on their photo-excited states [1]. The associated transient species are responsible for the bright fluorescence as well as the redox processes, which are desirable for energy conversion applications [37, 38]. However, a precise mechanistic pathway to account for the light-harvesting capability of the CDs is yet to be established. Nevertheless, recent research gives the impression of charge separation in the form of holes (radical cation) and electrons (radical anion) via photo-excitation of the core carbon nanoparticles of the CDs. The radiative recombination of these holes and electrons, which are trapped at various surface sites, results in the observed fluorescence emission [26]. Hence, to study the light-harvesting activity of the CDs, the photoluminescence behavior and HOMO–LUMO band-gap were evaluated.

**Fig. 4:**
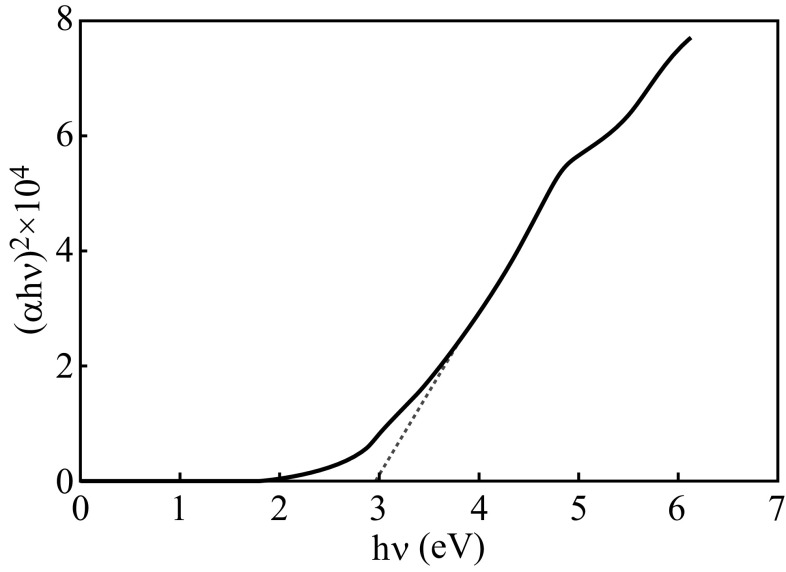
Plot of (*αhν*)^2^ versus *hν*

Like the signature behavior of other quantum dots, the CDs also exhibit excitation wavelength-dependent PL behavior (Fig. S2). Considering quinine sulfate as the reference, the quantum yield was calculated at the excitation wavelength of 360 nm and was found to be 9.5%. Values of 5.5, 6.7, 7.4, 7.8, and 7.6% were obtained for PNC1.0, PNC2.0, PNC3.0, PNC4.0, and PNC5.0, respectively. The band-gap was calculated from UV–visible spectroscopy by plotting *hν* versus (*α*h*ν*)^2^ (Tauc’s method) and was found to be 2.98 eV (Fig. 4). Here, it is pertinent to mention that the band-gap of carbon dots depends on the size and surface state of the nanoparticles, and the calculated value agreed with the literature data [39].

Generally, H_2_O splitting requires the bottom level of the conduction band to be more negative than the redox potential of H^+^/H_2_ (0 V vs. NHE), while the top level of the valence band should be more positive than the redox potential of H_2_O/O_2_ (1.23 V vs. NHE). Thus, the minimum value of *E*_bulk_ for any photo-catalyst should be 1.23 eV for H_2_O splitting. However, in practice, for effective H_2_O splitting, the band-gap should be larger than 2.0 eV. This is because, in reality, energy losses may occur due to kinetic overpotentials.

In the present study, *E*_bulk_ was evaluated as 2.98 eV for the CDs. Hence, these species satisfy the criteria to act as a catalyst for solar-driven production of H_2_O_2_. In this context, the CDs appear to be the superior catalyst compared to *g*-C_3_N_4_. This may be because, *g*-C_3_N_4_ has a band-gap of 1.4 eV, which is good enough to split alcohols but is insufficient to split H_2_O under solar irradiation. The photo-catalytic production of H_2_O_2_ involves photo-irradiation of O_2_-saturated H_2_O or alcohol in the presence of a photo-catalyst, i.e., the CDs in the present case.

Semiconductors can act as photo-catalysts for light-induced redox processes due to their electronic structure, which is characterized by a filled valence band and an empty conduction band. Absorption of a photon with energy greater than the band-gap energy leads to the formation of an electron/hole pair. In the absence of suitable scavengers, the stored energy is dissipated within a few nanoseconds by recombination. A plausible mechanistic framework involves the formation of an electron (e^−^)/hole (*h* ^+^) pair by photo-excitation of the CDs. In the case of an alcohol, h^+^ oxidizes the RCH_2_OH to generate aldehydes and a proton, while e^−^ promotes two-electron reduction of O_2_ to produce H_2_O_2_ [6, 21].

3 $${\text{CD}} \to {\text{e}}^{ - } + h^{ + }$$

4 $${\text{R}} - {\text{CH}}_{2} - {\text{OH}} + h^{ + } = {\text{R}} - {\text{CHO}} + 2{\text{H}}^{ + }$$

5 $${\text{O}}_{2} + 2{\text{H}}^{ + } + 2{\text{e}}^{ - } = {\text{H}}_{2} {\text{O}}_{2}$$

When H_2_O is used as the raw material, h^+^ oxidizes H_2_O to O_2_. Similar two-electron reduction of O_2_ follows thereafter to produce H_2_O_2_ [21].

6 $${\text{CD}} \to {\text{e}}^{ - } + h^{ + }$$

7 $$2{\text{H}}_{2} {\text{O}} + h^{ + } = {\text{O}}_{2} + 4{\text{H}}^{ + }$$

8 $${\text{O}}_{2} + 2{\text{H}}^{ + } + 2{\text{e}}^{ - } = {\text{H}}_{2} {\text{O}}_{2}$$

In the current study, we used H_2_O, mixtures of H_2_O and C_2_H_5_OH (25%, 50%, and 75% v/v), and C_2_H_5_OH (100%) saturated with molecular O_2_. In this regard, it is important to mention that many of the traditionally used semiconductors absorb only light energy or mostly in the UV region; hence, for a solar-driven process, dye sensitization is required [1]. However, the CDs can also absorb photons in the visible region; hence, the CDs can be readily used under sunlight. In the actual reaction setup, the reaction mixtures were irradiated under normal solar light with an intensity of 80,000–100,000 lx at ca. 25 °C for different durations.

**Fig. 5:**
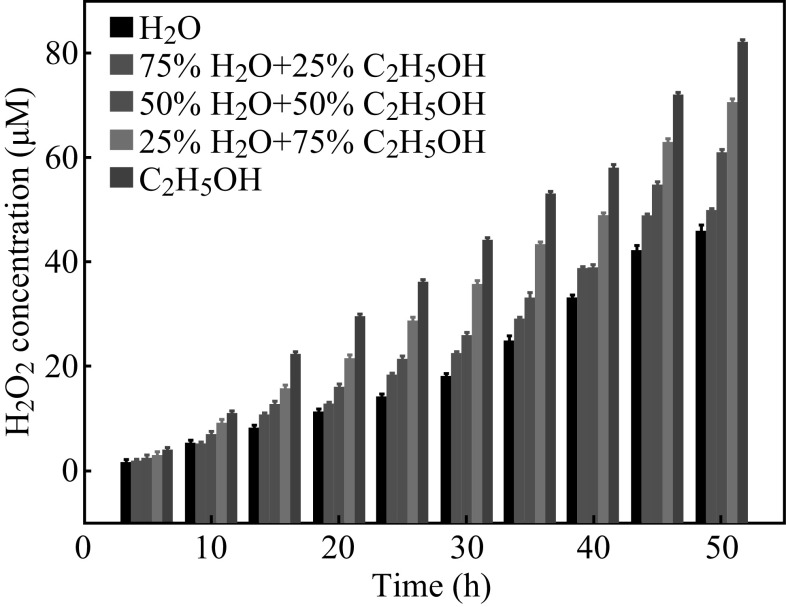
H_2_O_2_ production with different raw materials at different time intervals using PNC4.0 as catalyst

Figure 5 shows the formation of H_2_O_2_ at different intervals using different compositions of starting materials with PNC4.0 as the photo-catalyst. The bar diagram reveals the influence of the time and the composition of the starting materials on the production of H_2_O_2_. The formation of H_2_O_2_ increased with time for the photo-reaction. Here, it is pertinent to mention that the virtual yield of H_2_O_2_ shown in the bar diagram might be less than the actual amount formed, as some amount of H_2_O_2_ is destroyed by sunlight.

Nevertheless, the increased production of H_2_O_2_ with time clearly indicates the efficiency of the polymer-supported CDs as a photo-catalyst in the H_2_O splitting reaction. The rate of formation of H_2_O_2_ also supports this claim (Fig. S3). Some interesting observations emerge from this study. When H_2_O was used by itself, less H_2_O_2_ was produced (~46 µM after 50 h of reaction) compared to that obtained with 100% C_2_H_5_OH (~82 µM). On the other hand, in the case of the C_2_H_5_OH/H_2_O mixtures, the H_2_O_2_ production increased with an increase in the amount of C_2_H_5_OH in the mixture (~49 µM for 25%C_2_H_5_OH/75%H_2_O, ~61 µM for 50%C_2_H_5_OH/50%H_2_O, and ~70 µM for 75%C_2_H_5_OH/25%H_2_O). The increased H_2_O_2_ production in the presence of C_2_H_5_OH may be because of the favorable thermodynamic driving force, which is promoted by the suitable oxidation and reduction potential of the catalyst when alcohol is used (compared to water) [6]. A previous report also suggests similar behavior of alcohol and H_2_O mixtures toward solar-driven water splitting [21]. Thus, these data suggest that the photo-reaction of H_2_O with C_2_H_5_OH can enhance the H_2_O_2_ yield.

**Fig. 6:**
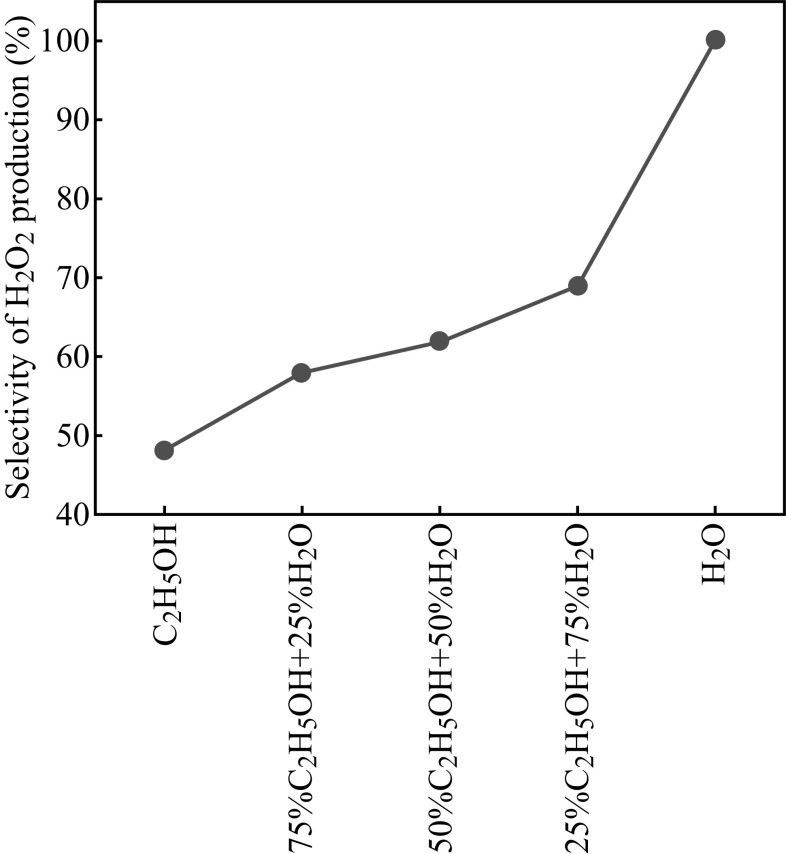
Selectivity curve for H_2_O_2_ production with different starting materials (using PNC4.0 as catalyst)

Figure 6 shows the selectivity toward H_2_O_2_ production when different compositions of starting materials were used. When H_2_O was used as the starting material, 100% selectivity for the production of H_2_O_2_ was achieved. However, when C_2_H_5_OH was used, the reaction showed no selectivity. Along with H_2_O_2_, a mixture of acetaldehyde and acetic acid was also formed, in which acetaldehyde was the predominant product, as confirmed by GC analysis. With an increase in the H_2_O gradient in the mixtures of C_2_H_5_OH/H_2_O, the selectivity toward H_2_O_2_ production also increased (~48% when only C_2_H_5_OH was used, ~58% for 75%C_2_H_5_OH/25%H_2_O, ~62% for 50%C_2_H_5_OH/50%H_2_O, ~69% for 25%C_2_H_5_OH/75%H_2_O, and 100% for H_2_O only). In other words, the production of H_2_O_2_ increased with respect to the by-products. This clearly suggests that the two-electron reduction of oxygen loses its selectivity when C_2_H_5_OH is used, whereas addition of H_2_O enhances the selectivity. Thus, it can be inferred that photo-reaction of C_2_H_5_OH with H_2_O around room temperature promotes selective H_2_O_2_ production.

**Fig. 7:**
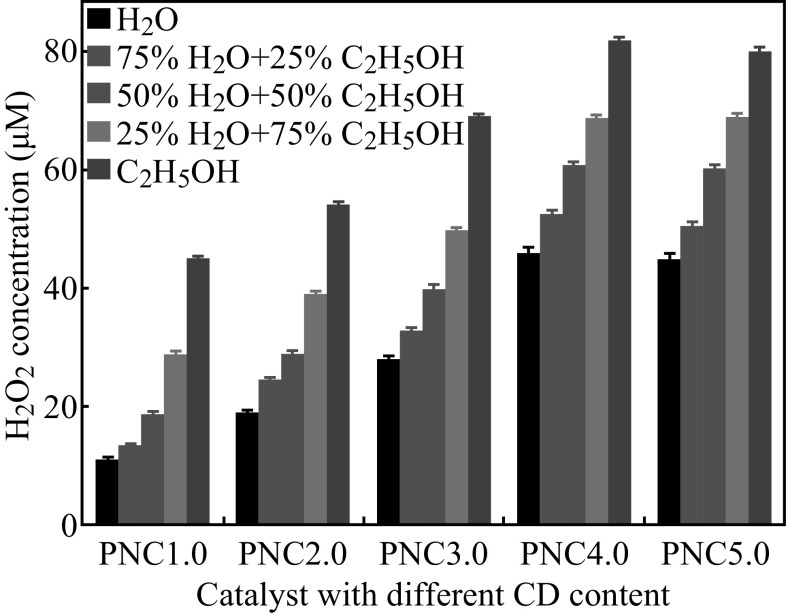
Effect of CD loading on H_2_O_2_ production (after 50 h of reaction)

The influence of the CD loading on H_2_O_2_ generation was evaluated (Fig. 7). In 50 h of photo-reaction, the production of H_2_O_2_ increased with an increase in the CD content up to 4 wt%. However, from a loading of 5 wt% onwards, the production of H_2_O_2_ declined slightly. This may be because of agglomeration of the CDs at higher loading, which reduces their light-harvesting ability. The quantum yields of the PNCs also supported this claim. PNC4.0 was found to have the optimal composition for H_2_O_2_ production via the photo-splitting of H_2_O and C_2_H_5_OH.

Here, it is pertinent to mention that CD alone can act as an effective catalyst with higher production yield (Fig. S4). However, the ease of separation makes the polymer-supported CDs more advantageous for use as a heterogeneous catalyst than the pristine CDs. This observation can be explained based on two factors. First, the increase in the size of the CDs after incorporation into the polymer matrix (due to intercalation of the polymeric chains in-between the layers of CD, which was confirmed by the increased *d*-spacing) may hamper the light-harvesting ability of the CDs. Secondly, the distribution of the CDs within the polymer matrix is not as uniform as in the solution state. This also contributed to reduction of the photo-catalytic activity of the polymer-supported CDs compared to the pristine CDs.

However, despite these facts, good photo-catalytic activity was observed for the polymer-supported CDs. The reusability of the CD-containing films was evaluated over several cycles. Reusability is an important factor for any catalyst from the green chemistry point of view. Accordingly, a model reaction was performed by using PNC4.0 as the photo-catalyst with a definite amount of reactants for 50 h. After completion of the reaction, the catalyst was recovered, dried, and used again under the same reaction conditions.

**Fig. 8:**
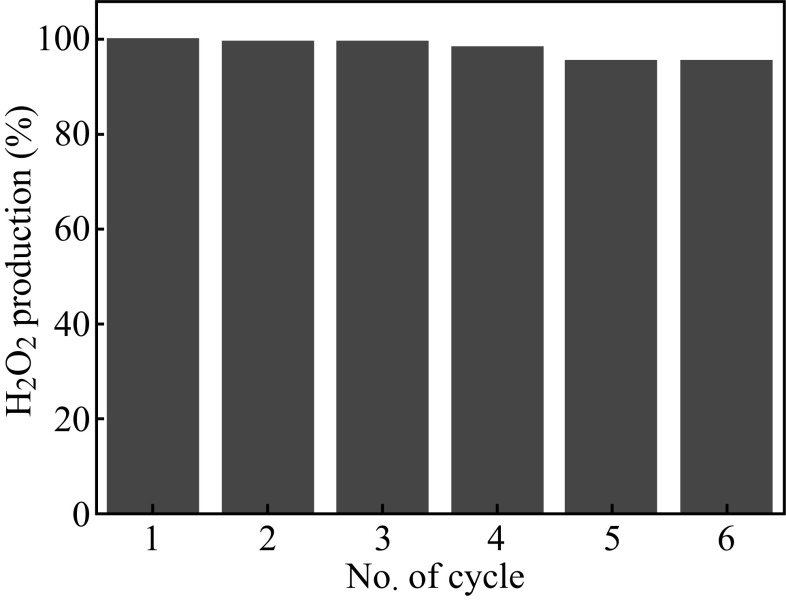
Reusability of the catalyst (PNC4.0)

Compared to the production of H_2_O_2_ (Fig. 8), there was no statistically significant difference up to the 4th cycle of use. This indicates the effectiveness of the polymer to hold CD within the matrix as well as to preserve its catalytic activity. Modification of the WPU/CD system with HE and PAA may contribute in this regard. Such modification results in a highly cross-linked system, which prevents leaching of the CDs from the polymer matrix. However, after the 5th cycle of use, a slight reduction in the yield was observed, though this reduction was not substantial. This may arise due to leaching of some amount of the CDs after several cycles of use, as was confirmed by UV–visible spectroscopy, where the spectrum of the reaction mixture showed a low-intensity peak near 270 nm (Fig. S5). Thus, the overall results confirm the utility of the polymer-supported CDs as a photo-catalyst for solar-driven production of H_2_O_2_ using O_2_, H_2_O, and C_2_H_5_OH as raw materials.

**Fig. 9:**
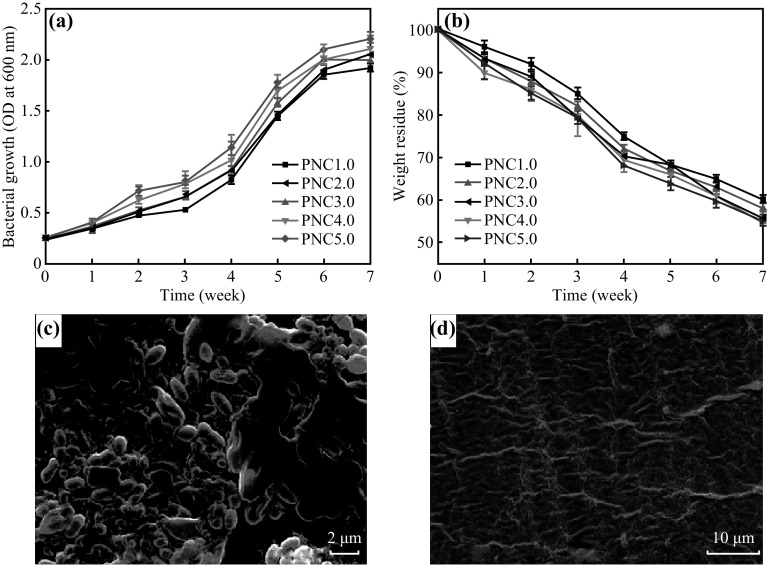
**a** Bacterial growth curves of PNCs. **b** Weight loss curves of PNCs. **c** SEM image of PNC4.0 after 7 weeks of bacterial exposure. **d** SEM image of reference film after 7 weeks

Bacterial degradation by *P. aureginosae* was evaluated to demonstrate the biodegradability of the catalyst system. Analysis of the bacterial degradation of PNC films revealed adequate biodegradation over the experimental period of seven weeks. The weight loss profiles confirmed that the WPU/CD nanocomposites offer a susceptible surface for bacterial adherence, resulting in high weight loss, which is almost consistent with the CD loading in the polymer matrix (Fig. 9a). The bacterial growth curves further support this observation (Fig. 9b). An increase in the bacterial population was recorded with time. With an increase in the CD loading, the rate of bacterial growth also increased. This is in contrast with the photo-catalytic activity of the PNCs, where the H_2_O_2_ production decreased when the CD loading exceeded 4 wt%. This is because *P. aeruginosa* is an organotroph microorganism that utilizes carbon-based materials as a source of food and energy. Hence, a high CD loading favors faster biodegradation. The SEM images (Fig. 9c, d) further confirmed significant bacterial adherence, as well as surface erosion, on the PNC films due to bacterial exposure. Thus, these results indicate the eco-friendly behavior of the catalyst system.

## Conclusion

From the above results, it can be inferred that carbon dots dispersed in a polyurethane matrix can be used as an effective heterogeneous photo-catalyst for solar-driven hydrogen peroxide production. The overall process can be justified as a green practice. It offers the benefits of green chemistry in terms of availability, facile and sustainable preparation, catalyst stability, non-toxicity, recyclability, and biodegradability. The key components, i.e., carbon dots, were synthesized using bio-based raw material in a cost-effective, facile process. Further, preparation of the polymeric support, i.e., the polyurethane matrix, was performed in an aqueous medium with a low VOC content. The reusability test confirmed the effectiveness of the catalyst, even after several cycles of use. Compared to other metal-based catalysts that are used for hydrogen peroxide production, the present system offers the benefits of non-toxicity and biodegradability. Further, compared to graphitic carbon nitride, the present system is cost-effective with the additional capability to produce hydrogen peroxide from water under sunlight. This study may pave the way for recognition of polymer-supported carbon dots as heterogeneous photo-catalysts.

## Electronic supplementary material

Below is the link to the electronic supplementary material.

Supplementary material 1 (PDF 779 kb)
