# Supplementary material for: Solar-Driven Hydrogen Peroxide Production Using Polymer-Supported Carbon Dots as Heterogeneous Catalyst
Source: Nanomicro Lett. 2017 Mar 17;9(4):40. doi: 10.1007/s40820-017-0143-7 (PMC6199036; doi:10.1007/s40820-017-0143-7)
Supplement: Supplementary file 1 — Supplementary material 1 (PDF 779 kb) [file 40820_2017_143_MOESM1_ESM.pdf]

Supporting Information for

Solar Driven Hydrogen Peroxide Production Using Polymer Supported Carbon Dot as Heterogeneous Catalyst

Satyabrat Gogoi[1], Niranjan Karak[1,*]

[1]Advanced Polymer & Nanomaterial Laboratory, Department of Chemical Sciences, Tezpur University, Assam, 784028, India

*Corresponding author. E-mail: nkarak@tezu.ernet.in

Tel: +91-3712-267009, Fax: +91-3712-26700

1 Preparation of CD Impregnated WPU

CD impregnated WPU was prepared according to the method as described elsewhere [1, 2, 3]. Briefly, isophorone diisocyanate (2.00 mol, Sigma-Aldrich, Germany), poly(ethylene glycol) with number average molecular weight 600 (0.80 mol, Merck, India), bis(hydroxyl methyl) propionic acid (0.53 mol, Sigma-Aldrich, Germany) were reacted in a four necked glass reactor equipped with a condenser, mechanical stirrer and nitrogen inlet. Reaction was carried out for 1.5 h in nitrogen atmosphere with stirring at a temperature of 85±2 °C. In the next step, 1,4-butane diol (0.54 mol, Merck, India) and tannic acid (0.07 mol, Sigma-Aldrich, Belgium) in limited amount of tetrahydrofuran (Merck, India) were added and the reaction was carried out within a close system at a temperature of 70±2 °C for 4-4.5 h. Then triethyl amine (0.53 mol, Merck, India) was added slowly at room temperature to create the ionic centers by neutralizing –COOH groups. After this step water was added at a very slow and steady rate with constant mechanical stirring. Then varying amount of CD (1-5 wt%) is incorporated to the polymer matrix by using mechanical stirring and ultra-sonication. Finally, tetrahydrofuran was removed carefully under reduced pressure. This polymeric system was cured with 20% glycerol based hyperbranched epoxy and fatty acid based poly(amido amine) using sound and heat energy [3]. Different amount of CD was used to obtain different catalyst systems using this standard preparative method.

2 UV-visible Spectra of CD and PNCs

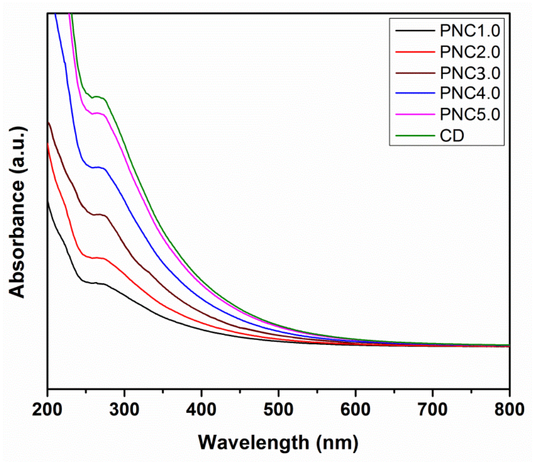

Fig. S1 UV-visible spectra of CD and PNCs

3 PL Spectra of PNCs

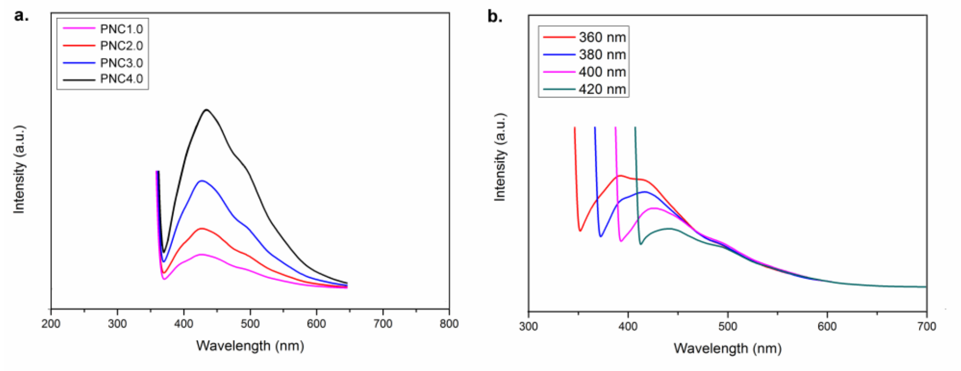

Fig. S2 a PL spectra of PNCs at excitation wavelength of 360 nm, **b** PL spectra of PNC4.0 at different excitation wavelengths

4 Rate of H_2O_2 Production

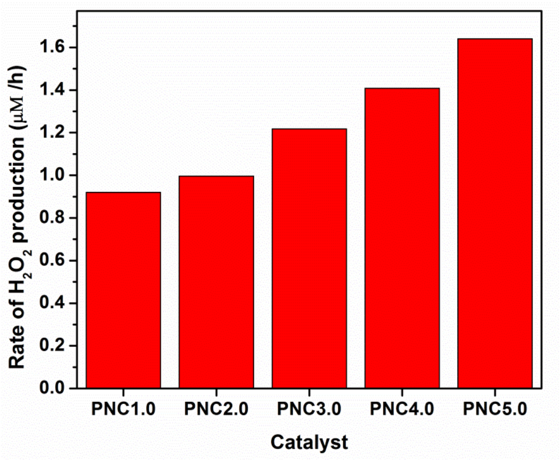

Fig. S3 Rate of H_2O_2 production by using PNC4.0 as the catalyst after a reaction of 50 h

5 Solar Driven H_2O_2 Production by Using CD as Catalyst

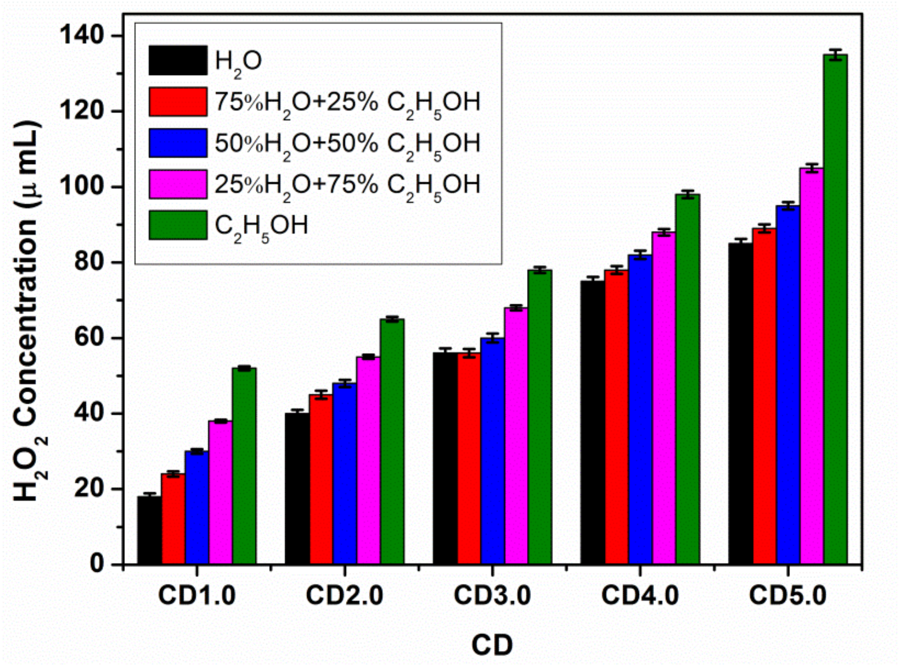

Fig. S4 Solar driven H_2O_2 production by using CD as the catalyst

6 Leaching Study of CD after 5th Cycle

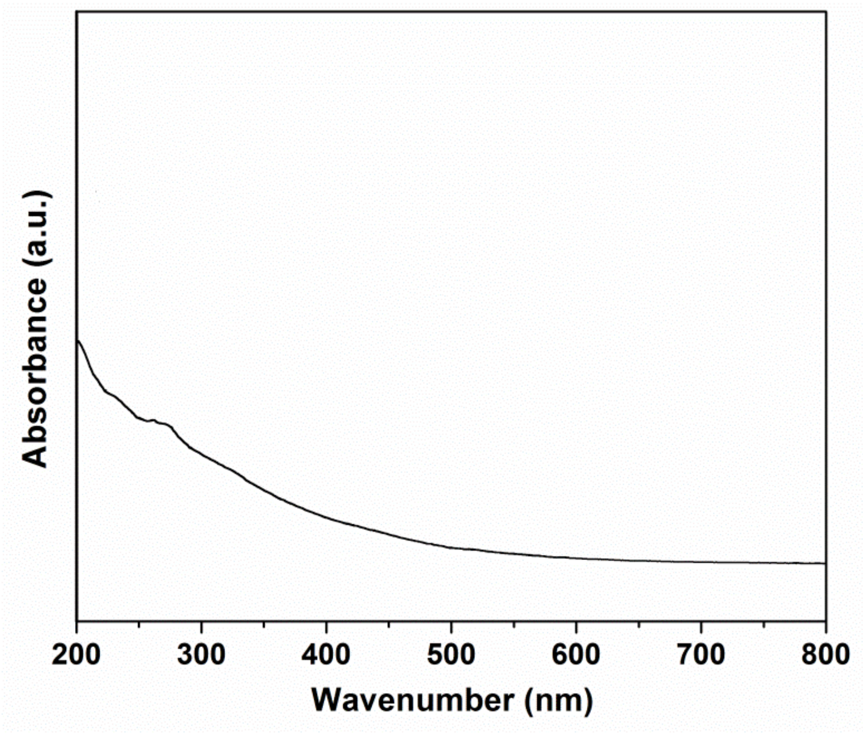

Fig. S5 UV-Visible spectrum of the reaction medium after 5th cycle

References

[1] S. Gogoi, N. Karak, Biobased biodegradable waterborne hyperbranched polyurethane as an ecofriendly sustainable material. ACS Sustainable Chem. Eng. **2**(12), 2730-2738 (2014). doi:10.1021/sc5006022

[2] S. Gogoi, N. Karak, Bio-based high-performance waterborne hyperbranched polyurethane thermoset. Polym. Adv. Technol. **26**(6), 589-596 (2015). doi:10.1002/pat.3490

[3] S. Gogoi, M. Kumar, B.B. Mandal, N. Karak, High performance luminescent thermosetting waterborne hyperbranched polyurethane/carbon quantum dot nanocomposite with invitro cytocompatibility. Comp. Sci. Technol. **118**, 39-46 (2015). doi:10.1016/j.compscitech.2015.08.010
